# S100A8/A9 Is Not Involved in Host Defense against Murine Urinary Tract Infection

**DOI:** 10.1371/journal.pone.0013394

**Published:** 2010-10-14

**Authors:** Mark C. Dessing, Loes M. Butter, Gwendoline J. Teske, Nike Claessen, Chris M. van der Loos, Thomas Vogl, Johannes Roth, Tom van der Poll, Sandrine Florquin, Jaklien C. Leemans

**Affiliations:** 1 Department of Pathology, Academic Medical Center, University of Amsterdam, Amsterdam, The Netherlands; 2 Institute of Immunology, University of Muenster, Muenster, Germany; 3 Center for Infection and Immunity Amsterdam and Center for Experimental and Molecular Medicine, Academic Medical Center, University of Amsterdam, Amsterdam, The Netherlands; University of Toronto, Canada

## Abstract

**Background:**

Inflammation is commonly followed by the release of endogenous proteins called danger associated molecular patterns (DAMPs) that are able to warn the host for eminent danger. S100A8/A9 subunits are DAMPs that belong to the S100 family of calcium binding proteins. S100A8/A9 complexes induce an inflammatory response and their expression correlates with disease severity in several inflammatory disorders. S100A8/A9 promote endotoxin- and *Escherichia (E.) coli*-induced sepsis showing its contribution in systemic infection. The role of S100A8/A9 during a local infection of the urinary tract system caused by *E. coli* remains unknown.

**Methodology/Principal Findings:**

We investigated the contribution of S100A8/A9 in acute urinary tract infection (UTI) by instilling 2 different doses of uropathogenic *E. coli* transurethrally in wild type (WT) and S100A9 knockout (KO) mice. Subsequently, we determined bacterial outgrowth, neutrophilic infiltrate and inflammatory mediators in bladder and kidney 24 and 48 hours later. UTI resulted in a substantial increase of S100A8/A9 protein in bladder and kidney tissue of WT mice. S100A9 KO mice displayed similar bacterial load in bladder or kidney homogenate compared to WT mice using 2 different doses at 2 different time points. S100A9 deficiency had little effect on the inflammatory responses to *E. Coli*-induced UTI infection, as assessed by myeloperoxidase activity in bladder and kidneys, histopathologic analysis, and renal and bladder cytokine concentrations.

**Conclusions:**

We show that despite high S100A8/A9 expression in bladder and kidney tissue upon UTI, S100A8/A9 does not contribute to an effective host response against *E. Coli* in the urinary tract system.

## Introduction

Most cases of “community-acquired” urinary tract infection (UTI) are due to enteric bacteria that enter the urinary tract, of which *Escherichia (E.) coli* is the most common organism (70–80%). UTI is a very frequent infection, affecting 12/10000 woman and 4/10000 men annually [1]. In the United States alone, the estimated annual societal cost of UTI is more than 3 billion dollars [2]. Moreover, UTI frequently occurs during childhood and may cause inflammation of the renal pelvis (pyelonephritis), which is a major factor in the development of end stage renal failure in children and young adults [3], [4]. Unfortunately, no licensed vaccine to prevent UTI exists, therefore unraveling the molecular mechanisms behind pathogen-host interaction is required for successful vaccine development. Bladder epithelial cells play an important part in the innate immune response during UTI because they express several Toll-like receptors (TLR), pattern recognition receptors which recognize motifs expressed by pathogens to induce an inflammatory response [5]. TLRs contribute to innate immune activation in the settings of both infection and sterile injury by responding to a variety of microbial, but also to endogenous proteins called danger associated molecular patterns (DAMPs) like heat shock proteins, high mobility group box chromosomal protein 1, heparan sulfate, hyaluronan fragments, and fibronectin [5], [6]. Although the manner by which pathogen-host interaction occurs during *E.coli*-induced UTI is slowly beginning to unravel [7], the contribution of DAMPs released upon inflammation is less clear. One group of the identified DAMPs are S100 proteins which mediate an inflammatory response and are involved in the recruitment of inflammatory cells to the site of infection [8], [9]. Of particular interest are S100A8 (myeloid-related protein MRP8; calgranulin A) and S100A9 (MRP14; calgranulin B). S100A8 and S100A9 form heterodimers, which are the biologically relevant forms of these proteins and have pleiotropic properties [9], [10], [11]. S100A8/A9 are found in granulocytes, monocytes and early differentiation stages of macrophages and can be induced in keratinocytes and epithelial cells under inflammatory conditions [9], [10]. S100A8 is almost not detectable at the protein level in mature phagocytes of S100A9 KO mice despite normal S100A8 mRNA levels, probably due to the elevated metabolism of S100A8 in the absence of its binding partner. Thus, targeted deletion of S100A9 leads to a complete lack of a functional S100A8/A9 complex in the mouse [12], [13]. In contrast to S100A8 KO mice, S100A9 KO mice are viable and in addition, lack the functional S100A8/A9 protein complex [13], [14]. Interestingly, S100A8/A9 protein complex interacts with TLR4 [12] and the extent of S100A8/A9 expression correlates with disease activity in several inflammatory disorders [9], [10], [15], [16]. So far, the knowledge about the contribution of S100A8/A9 during (urinary tract) infection is limited. Reyes *et al*. showed that *Ureaplasma parvum*-induced UTI lead to increased S100A8/A9 expression in bladder tissue however, its contribution was not further investigated [17]. Previously it was shown that S100A9 KO mice were protected against mortality induced by endotoxic shock and *E.coli* induced sepsis suggesting a detrimental role during systemic inflammation and infection [12]. This shows the contribution of S100A8/A9 in systemic infection, however its contribution in local infection is unknown. Therefore, we investigated the expression, localization and contribution of S100A8/A9 during *E. coli-*induced UTI.

## Materials and Methods

### Mice

Deletion of the MRP8/S100A8 gene in mice results in an embryonic lethal phenotype [18]. Therefore we used MRP14/S100A9 knockout (KO) mice which lack biological active S100A8/A9 protein complex [13], [14]. S100A9 KO mice were generated by target gene disruption of the *Mrp14* gene as described [13] and are backcrossed six times to a C57BL/6 background. Pathogen-free 9- to 10-week-old female C57BL/6 wild-type (WT) mice were purchased from Charles River Laboratories. Age-matched mice were used in all experiments. All mice were bred in the animal facility of the Academic Medical Center in Amsterdam, The Netherlands. The Animal Care and Use Committee of the University of Amsterdam approved all experiments.

### Urinary tract infection

Urinary tract infection (UTI) was induced as described earlier [19], [20], [21]. Briefly, *E. coli* 1677, an isolation from a patient with urinary tract infection, was grown overnight in Tryptone Soy Broth (TSB) medium at 37°C. The next day, a 1∶100 dilution in fresh TSB medium was grown until logarithmic phase, spun down and washed twice with cold phosphate-buffered saline (PBS). *E. coli* was resuspended in PBS and 10-fold serial dilutions were plated and grown on blood-agar plates overnight at 37°C to determine concentration. UTI was induced under general anesthesia with 0.07 mL/10 g mouse of FFM mixture, containing 1.25 mg/mL midazolam (Roche, Mijdrecht, The Netherlands), 0.08 mg/mL fentanyl citrate and 2.5 mg/mL fluanisone (Janssen Pharmaceutica, Beerse, Belgium). Hundred µl of bacterial suspension (total 4.5×10^8^ or 9.0×10^8^ colony forming units -CFUs-) was administered transurethrally through a 0.55 mm catheter (Abbott, Zwolle, The Netherlands). Mice were sacrificed 24 or 48 hours after the induction of UTI. Sham control mice underwent the same procedure with administration of 100 µL of sterile PBS and were sacrificed the following day.

### Bacterial outgrowth

Mice were anesthetized and heparin-blood and kidneys were collected. The left kidney from each mouse was weighted and homogenized in 4 volumes of sterile saline using a tissue homogenizer to correct for differences in weight (Polytron PT1300D homogenizer, Kinematica AG). The homogenizer was cleaned with 70% ethanol after each homogenization. The bladder from each mouse was homogenized in 9-fold volumes of saline. Serial 10-fold dilutions were made in sterile saline and 50 µL volumes of kidney homogenate, bladder homogenate and blood were plated onto blood agar plates, which were incubated at 37°C for 16 h, after which *E. coli* CFUs were counted. CFU count in urine was not performed due to technical difficulty; mice with UTI void their bladder more frequently making urine collection difficult.

### Preparation of tissue for ELISA measurements

#### For ELISA measurements, kidney homogenates were diluted 1∶2 in lysis buffer

(300 mM NaCl, 30 mM Tris, 2 mM MgCl_2_, 2 mM CaCl_2_, 1% Triton X-100, pepstatin A, leupeptin, and aprotinin, all 20 ng/ml; pH 7.4) and incubated at 4°C for 30 min. Homogenates were centrifuged at 1500 g and 4°C for 15 min, and supernatants were stored at −80°C until assays were performed. S100A8/A9 ELISA was performed as described earlier [12]. Tumor necrosis factor (TNF)-α, macrophage inflammatory protein (MIP)-2 and keratinocyte-derived chemokine (KC) were measured in kidney homogenate using specific ELISAs (R&D Systems) according to manufacturer instructions. Myeloperoxidase (MPO) was measured by ELISA (HyCult, Uden, the Netherlands).

### White blood cell count

White blood cell (WBC) counts in peripheral blood were determined using a hemocytometer (Beckman Coulter, Fullerton, CA, USA). For differentiation: 5 µl of whole blood was used for blood smear and stained with Giemsa (Diff-Quick; Baxter, McGraw Park, Il). Two-hundred to 250 WBC were counted at high power fields (magnification 400x) after which percentage of different cell types were determined.

### Immunohistochemistry

Right mouse kidney tissue samples were fixed in 4% formalin and processed to paraffin blocks. Four-micrometer sections were cut, mounted on coated slides and dried overnight at 37°C. After dewaxing and rehydration, heat-induced epitope retrieval (HIER) was performed by boiling tissue samples for 10 minutes in citrate buffer (pH 6.0). Immunohistochemical sequential double alkaline phosphatase (AP) staining was performed essentially as previously described [22]. Briefly, for granulocyte/S100A8-A9 double staining, sections were incubated overnight at 4°C with rat-anti-muis-Ly6G-FITC (BD Pharmingen, Erembodegem, Belgium) followed by rabbit anti-FITC antibody (Dako, Glostup, Denmark). For macrophage/S100A8-A9 double staining, sections were incubated with rabbit anti-rat F4/80. All slides were subsequently incubated with PowerVision anti-rabbit AP (ImmunoLogic, Duiven, The Netherlands). AP activity was visualized in blue, using Vector Blue (Vector Labs, Burlingame, CA, USA). Next, a second HIER step was applied to remove the antibodies from the first staining sequence, but leaving the deposits of blue reaction product unchanged [22]. Sections were incubated with rat-anti-mouse S100A8 or S100A9 (R&D systems, Abingdon, United Kingdom) for 3 hours followed by goat-anti-rat IgG AP. AP activity was visualized in red using Vector Red (Vector Labs). Antibodies were diluted in Antibody Diluent (ImmunoLogic, Duiven, The Netherlands). Spectral imaging was used to analyze colocalization of S100A8/A9 and with Ly6G or F4/80. Specimens were observed with a Leica BM15000 microscope (Leica Microsystems, Wetzlar, Germany) and analyzed with Nuance VIS-FL Multispectral Imaging System (Cambridge Research Instrumentation, Woburn, MA) with Nuance software version 2.10. Spectral data sets and spectral libraries of Vector Blue and Vector Red were acquired from 440–720 nm at 20 nm intervals [22], [23]. Single Ly6G staining was performed as described earlier [24].

### Statistical analysis

All data are presented as mean ± standard error of the mean (SEM) unless mentioned otherwise. Data were analyzed by the Mann–Whitney U-test. Difference in positive blood culture between groups was analyzed by Chi-square test. P<0.05 was considered to represent a statistically significant difference.

## Results

### S100A8/A9 expression and localization

**Figure 1:**
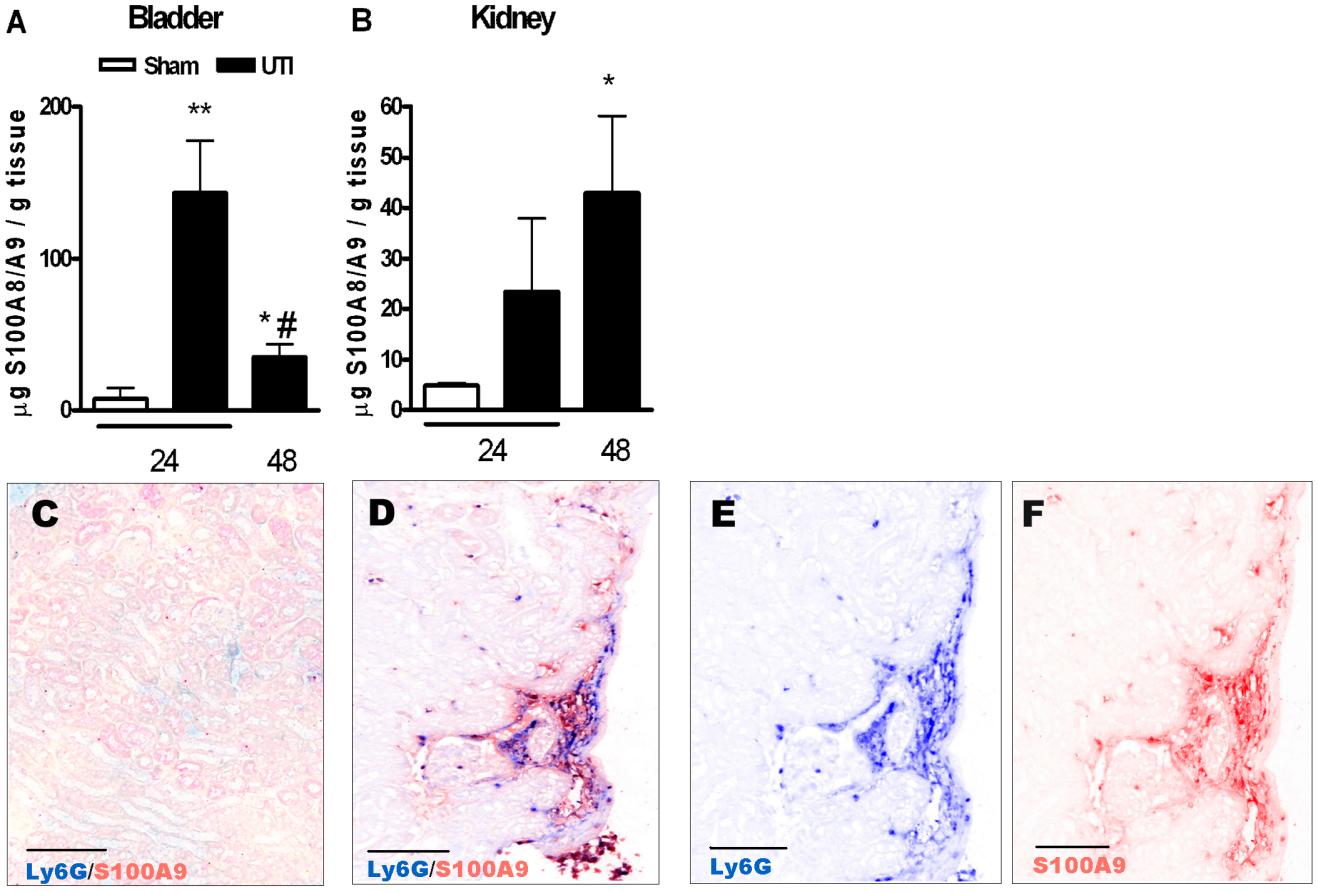
Expression and localization of S100A8/A9 during UTI. S100A8/A9 protein expression in bladder (1A) and kidney (1B) homogenate from sham WT mice (white bar) and from WT mice 24 and 48 hours after UTI with 9×10^8^ *E. coli* CFU/mouse (black bars). Data are mean ± SEM; N = 7–8 mice per group, *P<0.05, **P<0.005 versus sham, #P<0.005 versus 24 hours UTI. Sequential double AP staining using Ly6G (indicating granulocyte staining) and S100A9 antibodies on kidney tissue from sham WT mice (1C) or WT mice 24 hours after UTI with 9×10^8^ *E. coli* CFU (1D–F). Using spectral imaging Ly6G/S100A9 double staining was separately visualized for Ly6G (Vector Blue, 1E) or S100A9 (Vector Red, 1F). Scale bars display 0.1 mm.

First, we determined S100A8/A9 protein complex expression in bladder and kidney tissue from WT mice upon UTI induced by 9×10^8^ CFU/mouse. As shown in figure 1A–B, S100A8/A9 level was higher in bladder and kidney homogenates from WT mice 24 and 48 hours after induction of UTI compared to sham mice. S100A8/A9 protein expression was undetectable in kidney and bladder homogenate from naïve- or infected S100A9 KO mice (data not shown). To determine the localization and source of S100A8/A9 protein expression in kidney tissue, we performed double staining for S100A8/A9 with Ly6G (a marker for granulocytes) or with F4/80 (a marker for macrophages). Naïve WT mice displayed very few Ly6G, F4/80 or S100A8/A9 positive cells (Figure 1C and data not shown). Next, we performed double staining for Ly6G with S100A9 to study colocalization in kidney tissue slides obtained from WT mice, 24 hours after infection (Figure 1D–F). Figure 1D shows double AP staining indicating colocalization of Ly6G and S100A9. Using spectral imaging we could distinguish Ly6G positive cells (Vector Blue, figure 1E) from S100A9 positive cells (Vector Red, figure 1F). Double staining with Ly6G and S100A8 displayed similar patterns in kidney tissue slides as observed with Ly6G/S100A9 double staining (data not shown). F4/80-S100A9 double staining was absent in renal tissue slides obtained from WT mice 24 hours after infection (data not shown). As a control, S100A9 staining was absent in renal tissue slides obtained from S100A9 KO mice subjected to UTI (data not shown).

### Bacterial outgrowth

**Figure 2:**
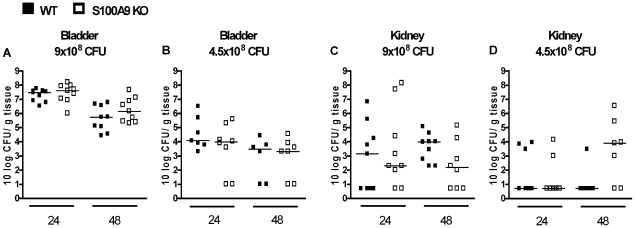
Contribution of S100A8/A9 to bacterial clearance during UTI. Bacterial outgrowth (CFU) in bladder (figure 2A+B) and kidney (figure 2C+D) homogenates from WT mice (black squares) and S100A9 KO mice (white squares), 24 and 48 hours after inoculation with 4.5×10^8^ or 9.0×10^8^ CFU *E. coli*/mouse. Single experimental data are depicted as individual squares, lines represent median. N = 6–9 mice per group.

S100A8/A9 is considered to have antimicrobial properties [25], [26], [27], [28], [29], [30], [31], [32], [33], [34]. To establish the role of S100A8/A9 in UTI, infection was induced in WT and S100A9 KO mice by instilling transurethrally 9×10^8^ CFU *E. coli*. Twenty-four and 48 hours thereafter, WT and S100A9 KO mice displayed similar bacterial loads in bladder and kidney homogenates (figure 2A+C). Dissemination of *E. coli* from the urinary tract to the circulation was not significantly different between WT and S100A9 KO mice 24 and 48 hours after infection (number of positive blood cultures; WT vs. S100A9 KO mice, t = 24: 0/9 vs. 3/9, P = 0.06 and t = 48: 0/9 vs. 0/9, P = 1.00). As S100A8/A9 might play a role during a less severe infection, we infected WT and S100A9 KO mice with 4.5×10^8^ CFU/mouse. Again, similar bacterial loads in the bladder and kidney were observed in WT and S100A9 KO mice 24 and 48 hours after infection (figure 2B+D). Dissemination of *E. coli* from the uropathogenic tract to the circulation was also undistinguishable in WT and S100A9 KO mice 24 and 48 hours after infection with 4.5×10^8^ CFU (number of positive blood cultures; WT vs. S100A9 KO mice, t = 24: 1/7 vs. 0/8, P = 0.27 and t = 48: 0/6 vs. 1/7, P = 0.34).

### Leukocyte recruitment

**Figure 3:**
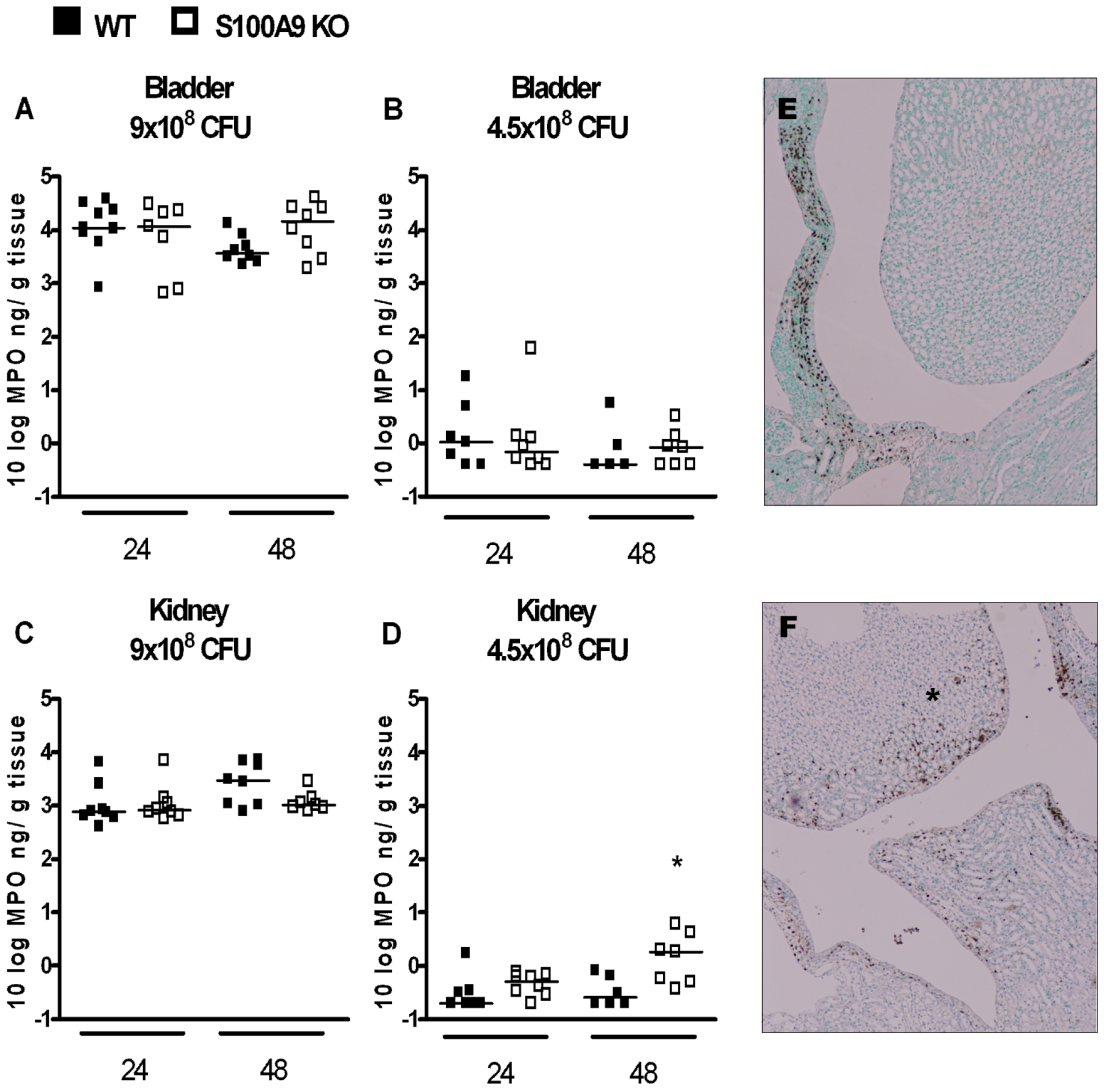
Contribution of S100A8/A9 to neutrophil influx during UTI. Granulocyte influx (as reflected by MPO levels) in bladder (figure 3A+B) and kidney (figure 3C+D) homogenates from WT mice (black squares) and S100A9 KO mice (white squares), 24 and 48 hours after inoculation with 4.5×10^8^ or 9.0×10^8^ CFU *E. coli*/mouse. Single experimental data are depicted as individual squares, lines represent median. N = 6–9 mice per group, *P<0.05 vs. WT mice. Representative picture of Ly6G staining from renal tissue slides obtained from WT mice (1E) and S100A9 KO mice (1F), 48 hours after infection with 4.5×10^8^ CFU *E. coli*/mouse. In S100A9 KO mice, more granulocytes were observed, especially in the renal pelvis (indicated by asterisk). Scale bars display 0.1 mm.

An essential part of the host defense against invading pathogens is the recruitment of neutrophils to the site of infection. Besides antimicrobial properties, S100A8/A9 has chemo-attractant properties [35], [36], [37], [38], [39]. We therefore determined levels of MPO to quantify neutrophil influx in both bladder and kidney homogenates (figure 3). MPO levels in bladder and kidney homogenate from sham mice were similar between WT and S100A9 KO mice (bladder: WT vs. S100A9 KO mice 0.21±0.03 vs. 0.38±0.15 ng/g tissue; kidney: WT vs. S100A9 KO mice 0.74±0.04 vs. 1.18±0.12 ng/g tissue, data are mean ± SEM, N = 7–8). Except for higher renal MPO level in S100A9 KO mice, 48 hours after instilling 4.5×10^8^ CFU (figure 3D), no differences in MPO level in bladder and kidney homogenate were observed between WT and S100A9 KO mice. The higher MPO level in S100A9 KO mice mentioned above was confirmed by Ly6G staining on renal tissue slides from WT and S100A9 KO mice, 48 hours after instilling 4.5x10^8^ CFU showing that S100A9 KO mice displayed more granulocytes in renal tissue, especially in renal pelvis (figure 3E+F). WBC counts and differentials in blood were similar in uninfected WT and S100A9 KO mice, as well as in infected WT and S100A9 KO mice at both time points using 2 different doses of *E. coli* (Table 1).

**Table 1:** White blood cell composition and total white blood cell count in blood from WT and S100A9 KO mice.

Sham		Mono (%)	Lympho (%)	Gran (%)	TCC (x 10^6^)
T24	WT	0.6±0.2	89.5±1.3	10.0±1.1	2.9±0.5
	S100A9 KO	0.7±0.2	88.6±2.1	10.7±2.1	4.0±0.7

9×10^8^ CFU		Mono (%)	Lympho (%)	Gran (%)	TCC (x 10^6^)
T24	WT	0.9±0.4	77.2±5.0	21.9±4.8	2.9±0.6
	S100A9 KO	1.0±0.4	73.0±5.4	25.9±5.2	4.0±0.7
T48	WT	0.6±0.2	76.5±2.7	22.9±2.7	5.7±0.8
	S100A9 KO	0.6±0.3	81.7±2.1	17.7±2.1	8.4±1.0

4.5×10^8^ CFU		Mono (%)	Lympho (%)	Gran (%)	TCC (x 10^6^)
T24	WT	1.6±0.3	82.0±2.8	16.3±2.6	10.8±1.3
	S100A9 KO	0.7±0.3	86.8±2.7	12.5±2.6	13.5±2.3
T48	WT	0.4±0.2	88.0±1.8	11.6±1.9	5.4±0.3
	S100A9 KO	0.5±0.1	84.1±3.1	15.4±3.2	4.5±0.7

White blood cell composition and total cell count in blood from WT and S100A9 KO mice, 24 and 48 hours after instillation with 9×10^8^ CFU uropathogenic *E. coli*/mouse or 4.5×10^8^ CFU/mouse. Sham mice were sacrificed 24 hours after inoculation with sterile PBS. Monocytes (mono), lymphocytes (lympho) and granulocytes (gran) are expressed as percentage of white blood cells. Total cell count (TCC) is presented as amount of white blood cells per ml. Data are mean ± SEM. (N = 6–8 per group).

### Inflammatory mediators

**Figure 4:**
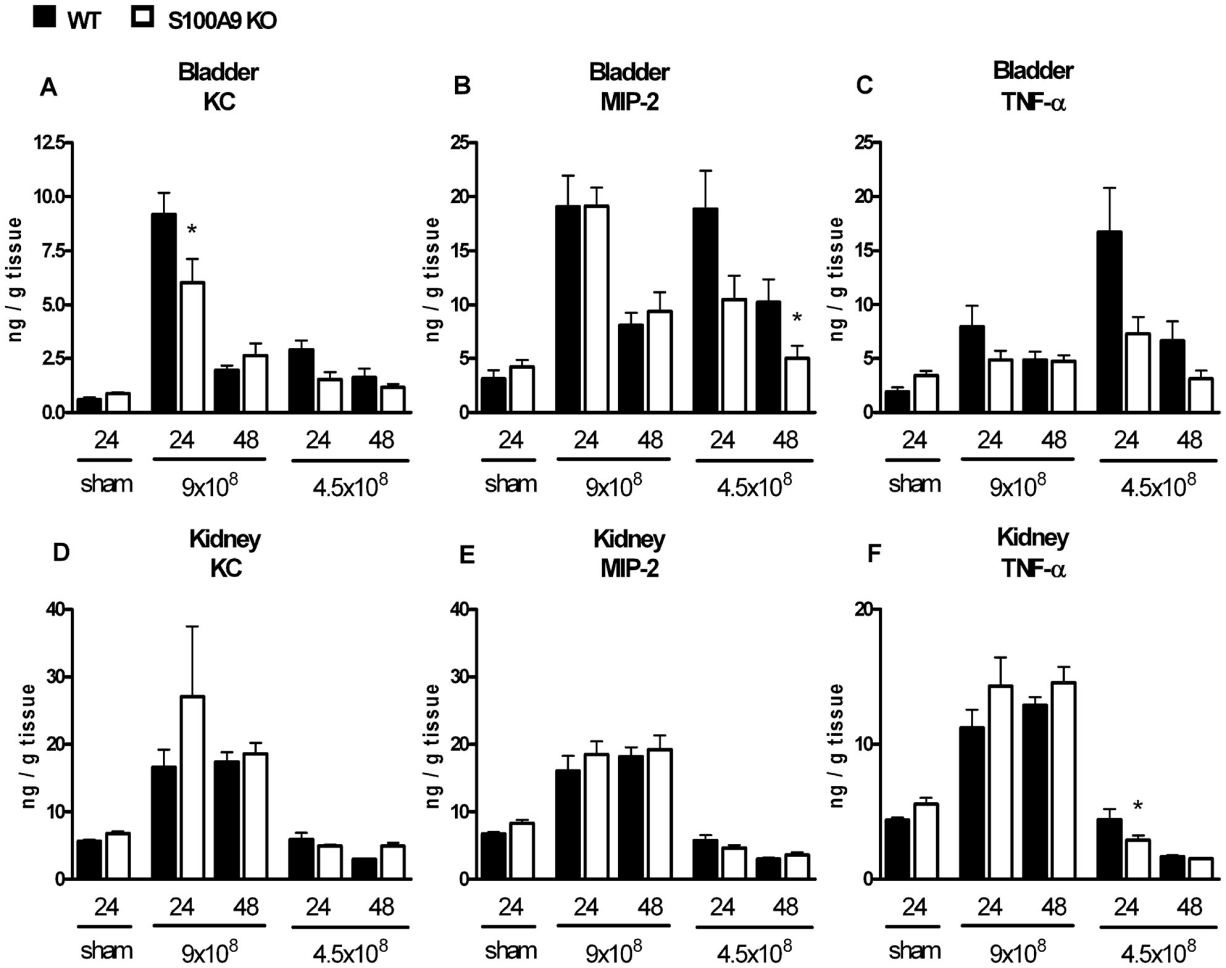
Contribution of S100A8/A9 to cytokine and chemokine production during UTI. Chemokine and cytokine levels (KC, MIP-2 and TNF-α) in bladder (figure 4A–C) and kidney (figure 4D–F) homogenates from WT mice (black squares) and S100A9 KO mice (white squares), 24 and 48 hours after inoculation with 4.5×10^8^ or 9.0×10^8^ CFU *E. coli*/mouse. Sham mice were sacrificed 24 hours after inoculation with sterile PBS. Single experimental data are mean ± SEM. N = 6–9 mice per group, *P<0.05 vs. WT mice.

Cytokines and chemokines play an important role in an adequate antibacterial defense. To determine whether S100A8/A9 deficiency affected production of inflammatory mediators, KC, MIP-2 and TNF-α were determined in bladder and kidney homogenates (figure 4). There were hardly any significant differences in cytokine and chemokine levels between WT and S100A9 KO mice. We only found significantly reduced KC and MIP-2 levels in bladder homogenates from S100A9 KO mice, respectively 24 hours after infection with 9×10^8^ CFU and 48 hours after infection with 4.5×10^8^ CFU. In kidney homogenates, only TNF-α levels were significantly reduced in S100A9 KO mice, 24 hours after infection with 4.5×10^8^ CFU.

## Discussion

UTI caused by uropathogenic *E.coli* is a substantial economical and social burden. Left untreated UTI can lead to pyelonephritis with increasing risk of permanent renal scarring and bacteremia. During inflammation, several DAMPs may be released by damaged host cells which can trigger the immune system to improve the clearance of infection or can contribute to inflammation-induced collateral tissue damage. One of these DAMPs, S100A8/A9 is highly expressed in bladder during complicated *U. parvum*-induced experimental UTI [17] and in serum of patients with sepsis caused by UTI [40]. Recently it was shown that S100A9 KO mice were protected against mortality during *E. coli*-induced sepsis[12] showing its contribution in systemic infection. This study is the first to investigate the expression, localization, and role of S100A9 in a local *E. coli*-induced infection of the urinary tract system. High levels of S100A8/A9 were observed in bladder and kidney tissue during UTI. To study the contribution of S100A8/A9 protein complex *in vivo* during UTI we used S100A9 KO mice which lack the functional S100A8/A9 protein complex. We found that the bacterial load in bladder or kidney homogenate of S100A9 KO mice was not significantly different from WT mice using 2 different bacterial doses at 2 different time points after infection. Moreover, S100A8/9 deficiency had little or no effect on the inflammatory response to *E. Coli*-induced UTI infection, as assessed by myeloperoxidase activity in bladder and kidneys, histopathologic analysis, and renal and bladder cytokine concentrations. In conclusion, S100A8/A9 deficiency did not influence the host response after infection with two doses of uropathogenic *E. Coli*, suggesting that endogenous S100A8/A9 does not play a major role in the pathogenesis of *E. Coli*-induced UTI.

Recently it has become clear that infection is commonly related to the release of DAMPs that serve to warn the host for eminent danger. S100 proteins have been suggested as DAMPs [12]. Of these S100 proteins, S100A8/A9 are of special interest because S100A8/A9 complexes induce an inflammatory response and S100A8/A9 expression correlates with disease severity in several inflammatory disorders [9], [15]. The expression of S100A8/A9 has been investigated in several infection models. S100A8/A9 levels were higher in lung tissue and bronchoalveolar lavage fluid during pneumococcal pneumonia in mice [39]. In addition, *U. parvum*-induced complicated UTI in rats, leads to higher expression of S100A8/A9 complex in bladder tissue compared to bladders from animals with asymptomatic UTI [17]. These data are in line with our results demonstrating that during UTI, levels of S100A8/A9 increased in both bladder and kidney homogenates. We anticipate that the S100A8/A9 expression we found originated from infiltrating granulocytes at infected areas. First, S100A8/A9 is the most abundant protein present in granulocytes [41]. Secondly, we found a strong colocalization of S100A8/A9 with granulocytes, but not with macrophages.

S100A8/A9 complexes 44also have antimicrobial properties [25], [26], [27], [28], [29], [30], [31], [32], [33], [34]. We however did not observe differences in bacterial outgrowth in kidney and bladder homogenate between WT and S100A9 KO mice at two different time points using two different doses of *E. coli*. In line with these results, Raquil *et al*. showed that pretreatment of mice with S100A8 and S100A9 antibodies, alone or in combination, had no effect on the bacterial load during pneumococcal pneumonia [39]. Our data suggests that during UTI, the lack of S100A8/A9 may be compensated for by other mediators. The previous study [39] and our data oppose a role for S100A8/A9 in antibacterial defense mechanism.

S100A8/A9 is known to have chemo-attractant properties [35], [36], [37], [38], [39]. Blocking S100A8/A9 inhibits neutrophil migration in response to LPS [42]. In addition, migration of granulocytes and macrophages to the alveoli was diminished during pneumococcal pneumonia when mice were treated with S100A8/A9 antibodies [39]. Expect for one particular time-point and *E.coli* dose in renal tissue, we observed no consistent difference in granulocyte influx in bladder and kidney tissue between infected S100A9 KO and WT mice. Our data argue against the specific chemoattractant properties of S100A8/A9 during *E. Coli*-induced UTI. Similar, except for several small differences in inflammatory mediators in kidney or bladder tissue between WT and S100A9 KO mice, we also did not observe large differences in production of inflammatory mediators.

Recently, the contribution of S100A8/A9 protein complex during infection has been appreciate again due to its pro-inflammatory properties and interaction with TLR4. So far, little is known about its expression, localization and contribution during infection *in vivo*. The novel S100A9 KO mice are a valuable tool in this research area. This study is the first to describe the contribution of S100A8/A9 during (*E.coli*-induced) UTI using S100A9 KO mice. We show that despite high S100A8/A9 expression in bladder and kidney tissue upon UTI, in this model S100A8/A9 does not contribute to an effective host response against *E. Coli* in the urinary tract system.
